# Some considerations on the current debate about typing resolution in solid organ transplantation

**DOI:** 10.1186/s13737-016-0032-5

**Published:** 2016-03-08

**Authors:** Paraskevi Vogiatzi

**Affiliations:** Department of Pathology, Tissue Typing Laboratory, University of Michigan, 2900 Huron Parkway, Ann Arbor, MI 48105 USA

**Keywords:** HLA typing, Antigen-based mismatch, Allele-based mismatch, Next generation sequencing, Solid organ transplantation, Cadaveric donor, Living donor

## Abstract

**Background:**

The shortage of suitable organs and achieved tolerance are uncontested main concerns in transplantation. Long waiting lists for deceased donors and limited numbers of living donors are the current scenarios. Kidney grafts from living donors have better overall survival compared to cadaveric and require less aggressive immunosuppressive regimens. The human leukocyte antigen (HLA) labs have the key role to test the recipient and donors compatibility based on typing and antibody profile. The current standard molecular procedure in solid organ transplantation is low-resolution typing, at the antigen level.

**Main text:**

In this commentary, the merits of high versus low degree of typing resolution in solid organ transplantation are discussed. Critical questions and reasons to bring high-resolution typing as a routine test in health system are considered. Specifically, with the introduction of the next-generation sequencing (NGS) in HLA, the pros and cons in living donation and benefits after deceased donation are critically evaluated.

**Conclusion:**

NGS has the potential to improve the transplant rates and the overall graft survival. Alternative strategies to increase in demanding the number of transplants are briefly highlighted.

## Background

In the kidney, the choice of living donor transplants offers better long-term outcomes than deceased organ transplants and the patients receive less aggressive immunosuppression [1]. Unfortunately, the living donors available are not sufficient to cover the urgent need of transplants and for other organs, such as the liver, pancreas, heart, lung, and the deceased donation path is unavoidable. The existent system of organ donation in the USA based on antigen-based mismatch acceptability, obtained from low-resolution typing of recipients and donors, is not working efficiently. For example, the number of active wait-listed kidney transplant candidates is over 75,000 in 2015. The average waiting time for a kidney transplant is about 5 years. Many patients are removed from the waiting list because they are too sick to undergo a transplant process, and around 4000 deaths per year are reported [2].

## Main text

Human leucocyte antigen (HLA) genes code for proteins playing a key role in immune responses and are notorious for being the most variable in the human genome. HLA gene sequences show high degree of polymorphism, not adequately captured by traditional typing tests, such as reverse sequence-specific oligonucleotide probes (SSOP), sequence-based typing (SBT), which is a Sanger sequencing reaction, and sequence-specific primers (SSP). For that reason, a committee from the American Society for Histocompatibility and Immunogenetics (ASHI) focused on a list of common and well-documented (CWD) HLA alleles, with a frequency greater than 0.001 in reference populations of at least 1500 individuals and reported more than three times in unrelated individuals, respectively, and rare alleles as well [3–5]. Many of these alleles have only been partially characterized until now. The DNA sequence of these incomplete alleles, as published in the International ImMunoGeneTics project (IMGT)/HLA database, is most often limited to exons that code for the extracellular domains of the mature protein. The above mentioned tests often result in ambiguities since they provide only segments of HLA genes and fail to distinguish among different alleles suggested by a given sequence and/or define polymorphisms lying outside the amplified region. Furthermore, preliminary molecular testing is often followed by a second level of reflexive and confirmatory typing, increasing costs and time.

**Fig. 1:**
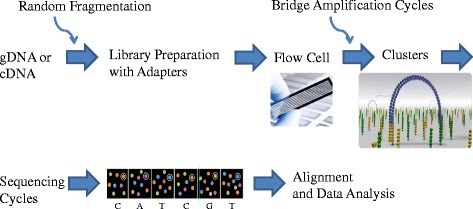
An overview of DNA sequencing by synthesis technology (Illumina). After random fragmentation of genomic or cDNA, adapters, which are specialized oligonucleotides, are bound to both 5**′** and 3**′** DNA fragment ends, allowing ligation and NGS library preparation. The library is loaded into a flow cell that is a glass slide with one (for MiSeq technology) or eight lanes (for HiSeq, allowing eight independent experiments) coated with surface-bound, adapter-complimentary oligos. The sample is hybridized onto the flow cell, generating a cluster and is clonally amplified through bridge amplification until the cluster has 1000 copies. Each cluster on the flow cell produces a single sequence read. The flow cell is imaged, and the emission from each image is recorded. Finally, the reads are aligned to a reference sequence using bioinformatics tools

In order to achieve allele information, a higher degree of resolution is offered by a combination of conventional SBT and also SSP to resolve ambiguities and/or confirm rare alleles. The highest possible resolution, covering the full HLA genomic region, is provided by various next-generation sequencing (NGS) platforms, which are available in the market right now, such as Roche GS 454 FLX, Ion Torrent PGM, Illumina MiSeq/HiSeq (Fig. 1), and Pacific Biosciences SMRT (Table 1). The MiSeq platform offers higher resolution HLA typing results, faster, less expensive, and easier work flow compared to Sanger and other NGS tools. The overall turnaround time is very comparable between Ion Torrent, MiSeq, and PacBio. The complexity of sample preparation is higher with PGM and PacBio, while the actual sequencing time is longer with MiSeq. PacBio is an excellent alternative technology to MiSeq with long read lengths but the equipment is very expensive. By the application of the NGS platforms, the list of HLA alleles increases dramatically [6, 7]. In the bone marrow work-up, because of the high cost of using high-resolution typing methodologies, the donors are typed at low- or intermediate-resolution SSOP, with a repeat, high-resolution SBT, SSP testing to assess compatibility for best recipient-donor pairs before the transplant process. With the above NGS methods, no additional typing will be necessary to assess compatibility since the donors are typed at the allele level from the very beginning.

**Table 1:**
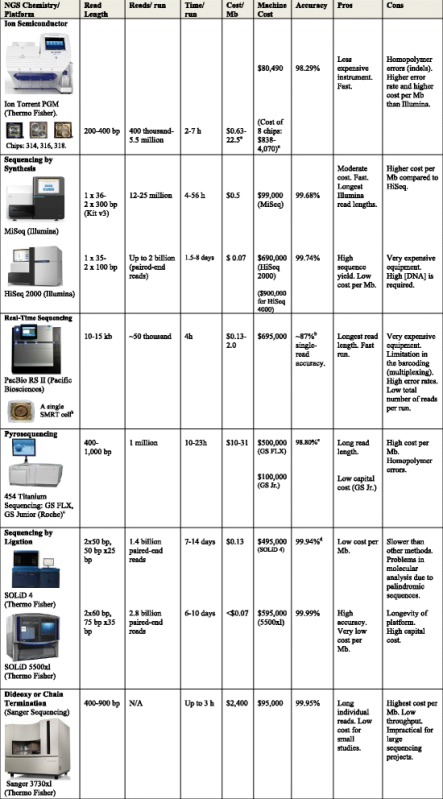
Comparison of the most common NGS tools and Sanger sequencing

The cost analysis is presented in US dollars. List pricing may vary between countries and/or sales territories

*N/A* not applicable

^a^Cost depending on the chip used, i.e., the 314 offers the lowest price ($838) but has the most expensive run ($22.5)

^b^The PacBio uses a chip called SMRT or Single-Molecule Real-Time. The single-read accuracy is ~87 %, whereas the consensus accuracy has been seen at 99.999 %

^c^The 454 sequencing platform will not be supported after 2016. The consensus accuracy is 99.997 % estimated at 15× coverage of *E. coli*

^d^SOLiD4: 99.999 % consensus accuracy at 15× coverage

Duquesnoy et al. discuss the positive impact of high-resolution typing in highly sensitized patients awaiting living donor transplants [8]. This important publication voices the opinion of academic clinicians, lab directors, and transplant surgeons in the USA, Canada, and the UK that emphasize the benefits of high-resolution HLA typing. The use of low-resolution typing in solid organ transplant candidates is not efficient when we handle situations, such as patients with antibody profile with a broad shared epitope Bw4 or in cases with mismatches of alleles that are not present in the Luminex single antigen bead (SAB) panel. In order to find the suitable donor for a recipient, epitope analysis becomes necessary to identify the amino acid structure corresponding to the epitope of alleles not found in SAB. Many times, we also have to run different vendors’ tests in parallel to better characterize or confirm the existence of HLA-DR, HLA-DQ, and HLA-DP antibodies.

On the other hand, another conservative approach from experienced HLA laboratory directors is against the use of high-resolution typing methods as routine use in solid organ transplantation because they believe it is more expensive than a low-resolution typing/virtual crossmatch and will block the organ offers preemptively [9]. The waiting list for available organs is long, even longer if the patients are highly sensitized, and adding more complexity with high-resolution typing tests is not a priority and will not be helpful for the patients.

The debate is especially important since it revolves on what is beneficial for the transplant candidates. Reliance on antigen-based mismatch is indicated in ethnically homogeneous populations, but since in the USA this is seldom the case, allele-based information by high-resolution typing is recommended. Epitope fine analysis could offer important insight in the case of rare alleles and/or alleles, not present in SAB panel, which share epitope(s) with common alleles. The use of tables with haplotype frequencies in various populations like those available from National Marrow Donor Program (NMDP) is also necessary for other transplants. Furthermore, the calculated Panel Reactive Antibody (cPRA) in the USA, which is essential for organ allocation, does not include DQA, DPA, and DPB antigens in contrast with the Canadian cPRA [10]. The current cPRA in the USA underestimates the unacceptable antigens reported in United Network for Organ Sharing (UNOS), misguiding selection of appropriate donors. High-resolution typing of the candidates for solid organ transplantation will provide sufficient information for all HLA loci helping in the generation of accurate critical alert systems at the transplant centers, if needed, when impossible to include in UNOS. It will decrease the errors in donor selection process and will favor the correct interpretation of unexpected positive crossmatches.

At this point, to assess donor-recipient compatibility in both bone marrow and solid organ transplantation, I would encourage the use of the best tool available for high-resolution typing, i.e., next-generation sequencing. It is true that NGS provides an enormous amount of genetic information; hence, the HLA clinical labs need to train the medical technologists investing on a new technology, hire biostatisticians, and pass strict validation procedures and the accreditation process. Nonetheless, effective analysis and interpretation through NGS will offer a complete, unambiguous, highest degree resolution typing of our patients. Overall, a complete genomic characterization of new HLA alleles and complete sequence of the existing, though so far only partially sequenced, alleles will be obtained.

NGS typing for recipients through hematopoietic stem cell and living solid organ transplantations is available in about 3 days. NGS technology is faster than the combination of traditional tests (SSOP, SBT, and SSP) till now used and confirmatory typing requested for the final pair recipient-donor in bone marrow field. It produces unambiguous results with no need or lesser need to report NMDP coding. Currently, the above technologies are cost-equivalent but NGS would be more robust and also cheaper if more samples are run (Table 1). A highly sensitized patient awaiting a living kidney donor needs accurate information for better selection of organ aiming at a better clinical outcome. Unsensitized pre-transplant renal recipient may not consider NGS testing although it can be a useful post-transplant, i.e., with the presence of de novo anti-HLA class II donor-specific antibodies (DSA) [11].

For transplants of organs from deceased donors, quick typing of donor samples takes place at the local Organ Procurement Organizations (OPO) using real-time PCR or frozen prepared reverse SSOP trays, and high-resolution typing is usually unavailable [12]. This causes uncertainty in reporting DSA and virtual crossmatching. Post-transplant NGS still can be retrospectively useful if questions are raised to identify missed DSA in a graft dysfunction, interpret biopsy data, better manage the recipient, avoid infectious complications, and evaluate the possibility of another living or deceased donor transplant.

## Conclusion

Allele-based mismatch acceptability is a keystone of solid organ transplantation immunology. This task can now take advantage of high-resolution typing. The focus of this commentary is specifically the application of NGS technology in clinical setting. While providing an overwhelming amount of information for the patient and donors, NGS will fill the gaps in HLA genomic regions that were previously uncharacterized. Its routine use in healthcare would benefit our patients finding suitable living donors with a single technology, in a single run, and with more chance of long-term success for the transplant. Until NGS is able to provide data in few hours, it will not be used for deceased donor selection. This means that the other typing methods will continue to be used, when needed, and nothing will be discarded. The laboratory director has discretion to judge the need for NGS or Sanger sequencing and make different decisions based on the patient’s degree of sensitization and the solid organ (i.e., show flexibility to candidate seeking a simultaneous liver/kidney transplant with HLA class I antibodies) [13]. In any case, I would suggest, for the labs that will not adopt the NGS technology immediately, that the lab director and the supervisors begin a self-education process. Furthermore, the requested rigorous validation studies for NGS have discouraged many labs so far. It will be helpful if the companies providing NGS platforms further optimize their products and if the accreditation committees become more collaborative. In the era of personalized medicine, NGS will help understand how specific individuals respond to infectious diseases, to vaccinations, and to particular immunosuppressive drugs.

The transplantation process should be seen in various directions: ABO blood group incompatible transplants for recipients of less than 24 months, dual kidney en bloc transplants from pediatric deceased donors, domino transplants, minimally invasive robotic surgery transplants for obese patients with body mass index (BMI) over 35 previously denied transplant, as well as the use of rejection prediction from assays, such as the Kidney Solid Organ Response Test (kSORT) [14–17]. Early involvement of the 58 federally supported OPOs in the USA is essential in organ transplantation with offers from cadaveric donors. In addition, an appropriate medical management of the deceased donors would increase significantly the donation opportunities. Since the live donor transplantation is the best treatment offer, strong educational programs led by highly committed physicians, coordinators, and advocates could help remove misconceptions about the living donation [18].

This is the time to view transplantation issues with new lenses and invest in novel technologies, combining efforts of transplant teams to increase organ availability and suitability. The contribution of the next-generation sequencing in the donor selection process should not be ignored.

## Abbreviations

ASHI: American Society for Histocompatibility and Immunogenetics
BMI: body mass index
CWD: common and well-documented
DSA: donor-specific antibodies
HLA: human leukocyte antigen
IMGT: International ImMunoGeneTics project
kSORT: Kidney Solid Organ Response Test
NGS: next-generation sequencing
NMDP: national marrow donor program
OPO: Organ Procurement Organization
SAB: single antigen beads
SBT: sequence-based typing
SSOP: sequence-specific oligonucleotide probes
SSP: sequence-specific primers
UNOS: United Network for Organ Sharing
